# Simvastatin modulates sleep–wake behavior and locomotor activity in a dose-dependent manner in *Drosophila*

**DOI:** 10.3389/fphar.2026.1815042

**Published:** 2026-07-01

**Authors:** Ahmed M. Alsehli

**Affiliations:** 1 Department of Physiology, Faculty of Medicine, King Abdulaziz University, Jeddah, Saudi Arabia; 2 Functional Pharmacology and Neuroscience, Department of Surgical Sciences, Uppsala University, Uppsala, Sweden

**Keywords:** behavioral pharmacology, *Drosophila*, locomotor activity, simvastatin, sleep regulation

## Abstract

**Background:**

Statins, including simvastatin, are widely prescribed cholesterol-lowering drugs with well-established cardiovascular benefits, yet their potential neurobehavioral effects, including sleep disturbances, remain unclear. Because sleep regulation in *Drosophila melanogaster* is conserved with mammals, this model provides a powerful system to investigate drug-induced behavioral changes. Given the reported neurobehavioral effects of lipophilic statins, we hypothesized that simvastatin exposure would alter sleep–wake behavior and sleep architecture in *D. melanogaster*.

**Methods:**

Wild-type flies were treated with simvastatin at concentrations of 0.00 (control), 0.05, 0.5, and 1.0 mM, and behavioral phenotypes were quantified.

**Results:**

We observed a disruption of sleep architecture: 0.05 mM produced no detectable effect; 0.5 mM induced a robust insomnia-like phenotype characterized by reduced total sleep and prolonged sleep latency; whereas 1.0 mM increased arousal, enhancing wake activity and delaying sleep onset despite preserved total sleep amount.

**Conclusion:**

These findings demonstrate that simvastatin exerts distinct, dose-dependent effects on sleep regulation, differentially affecting sleep initiation and maintenance. Collectively, this study suggests that *Drosophila* may serve as a useful model for investigating the behavioral effects of simvastatin, although further studies are required to validate and extend these observations.

## Graphical Abstract

**Figure:**
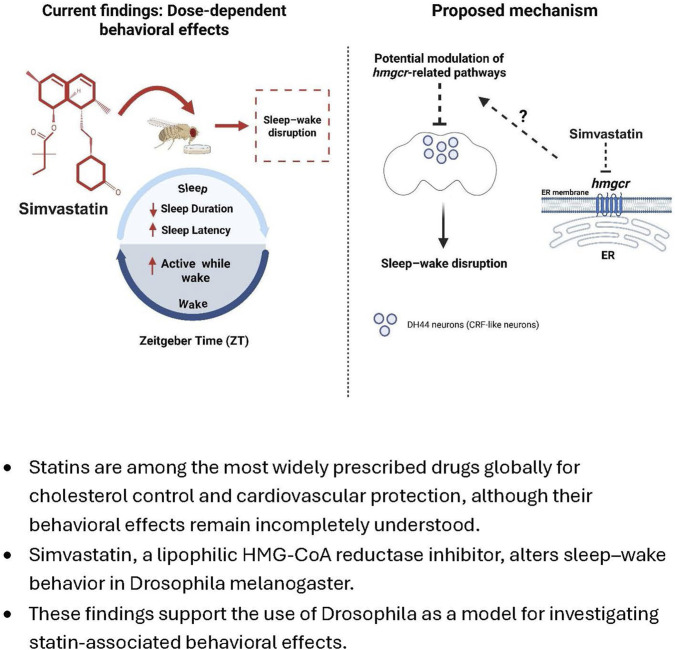

## Introduction

Sleep coordinates metabolic (Direksunthorn, 2025), cognitive (Walker, 2009; Rasch and Born, 2013), emotional (Walker, 2009), and cardiovascular functions (Cappuccio and Miller, 2017) and is therefore subject to tight biological control. Central to this control is sleep homeostasis, which maintains the balance between sleep and wakefulness, yet the biological and biophysical mechanisms and substrates through which homeostasis is implemented remain incompletely defined (Deboer, 2018; Eban-Rothschild et al., 2018; Allada et al., 2017). Prolonged wakefulness is accompanied by widespread alterations in brain metabolism (Reich et al., 1972; Porkka-Heiskanen et al., 1997), gene expression (Cirelli et al., 2004; Dopp et al., 2024), cellular architecture (de Vivo et al., 2016; Aboufares El Alaoui et al., 2023), synaptic connectivity (Tononi and Cirelli, 2014), and neuronal activity (Steriade et al., 2001; Vyazovskiy et al., 2009), underscoring the complexity of the processes that regulate sleep need. Perturbation of these regulatory processes can manifest as sleep disturbances, including insomnia, the most prevalent sleep disorder worldwide, affecting approximately one-third of adults (Morin and Jarrin, 2022) and increasing markedly with age (Nguyen et al., 2019). Clinically, insomnia is characterized by difficulty initiating or maintaining sleep, or by early morning awakening (Seugnet et al., 2009). Beyond disrupted sleep, insomnia is associated with elevated risks of cardiometabolic disease (Sofi et al., 2014), cognitive impairment (Fortier-Brochu and Morin, 2014; Fulda and Schulz, 2001) and substantial reductions in daytime functioning and quality of life (Morin et al., 2015; Riedel and Lichstein, 2000) and shows a bidirectional relationship with psychiatric disorders (Alvaro et al., 2013; Abu Khait et al., 2025). Despite its prevalence and clinical significance, the mechanistic basis of insomnia, and more broadly of sleep disturbances and their modulation by pharmacological interventions are still poorly understood.

*Drosophila melanogaster* is a powerful genetic and pharmacological model for dissecting the interactions between molecular pathways, drugs, and behavior, particularly in the regulation of the sleep–wake cycle (Agosto et al., 2008; Tomita et al., 2017). Core molecular pathways and sleep homeostatic mechanisms are highly conserved between *Drosophila* and mammals, making the fly an established system for mechanistic sleep research (Beckwith and French, 2019). Numerous genes, neural circuits, and biological processes that govern sleep regulation have been identified (Shafer and Keene, 2021). In brief, sleep is an evolutionarily conserved behavioral state characterized by prolonged periods of reduced activity and quiescence (Hendricks et al., 2000; Shaw et al., 2000). In fruit flies, like mammals, sleep consists of sustained episodes of behavioral quiescence that are regulated by both circadian and homeostatic mechanisms and emerge from modulation of locomotor output in distinct localized regions of the fly brain, including hypothalamus-like center (Cavanaugh et al., 2014; Sitaraman et al., 2015). Under standard 12:12 h light–dark conditions, flies exhibit a characteristic bimodal activity pattern, with peaks in the early morning and evening separated by a midday rest period (siesta) and followed by consolidated nocturnal sleep.

Statins are cholesterol-lowering agents that act by competitively inhibiting the enzyme 3-hydroxy-3-methylglutaryl-CoA reductase (HMG-CoA reductase, HMGCR), the rate-limiting enzyme in the mevalonate pathway for cholesterol biosynthesis (Maron et al., 2000). They represent an important class of therapeutics and are among the most widely prescribed drugs worldwide, with an estimated 200 million users globally; in the United States alone, approximately one in four adults over the age of 40 takes a statin, contributing to annual sales in the hundreds of billions of dollars (Jamshidnejad-Tosaramandani et al., 2022; Blais et al., 2021; Newman et al., 2019), largely due to their protective role in both primary and secondary prevention of cardiovascular disease (Chou et al., 2022; Paparodis et al., 2025; Delahoy et al., 2009).

Several statin subclasses exist, differing in potency, pharmacological properties, efficacy, and adverse effect profiles, influencing both therapeutic outcomes and tolerability (Chong et al., 2001; Gencer and Mach, 2018). Simvastatin, a lipophilic statin, remains one of the most prescribed agents globally and is frequently reported as among the leading drugs within its class, reflecting its long-standing clinical use and proven efficacy (Lippi et al., 2019). Owing to their higher capacity to cross the blood–brain barrier, lipophilic statins are generally considered more likely than hydrophilic statins to exert effects on the central nervous system (Ward et al., 2019). Despite their well-established benefits in reducing hyperlipidemia, statins can still produce pleiotropic side effects that may lead to treatment discontinuation (Ruscica et al., 2023). Some studies, including observational studies and post-marketing surveillance, have reported associations between statin therapy and sleep-related disturbances, including insomnia, reduced sleep efficiency, vivid dreams, and increased nocturnal awakening (Broncel et al., 2015; Cham et al., 2009; Szmyd et al., 2021; Takada et al., 2014; Pop et al., 2022). However, findings across studies have been inconsistent, with other reports showing no significant effects on sleep parameters. Therefore, a causal relationship remains unproven, and the overall impact of statins on sleep quality remains unclear. These inconsistencies may reflect differences in study design, population characteristics, and the limited number of trials specifically designed to assess sleep outcomes. In addition, it remains unresolved whether any observed effects are mediated by direct HMGCR inhibition or by cholesterol-independent (pleiotropic) mechanisms, including alterations in isoprenoid intermediates and downstream G-protein signalling (Swerdlow et al., 2015; ; Liao and Laufs, 2005). Previous work demonstrated that pharmacological inhibition of HMG-CoA reductase by fluvastatin alters sleep–wake behavior and sleep architecture in *Drosophila* (Alsehli et al., 2022). In that study, fluvastatin exposure increased sleep duration and reduced sleep latency, producing a phenotype similar to neuronal *hmgcr* knockdown in insulin-producing cells (IPCs) within the pars intercerebralis (PI), a hypothalamus-like structure in the fly brain. In contrast, *hmgcr* knockdown in another PI neuronal population, the *DH44* neurons, the *Drosophila* corticotropin-releasing factor (CRF) homologue-expressing neurons, produced a distinct phenotype characterized by reduced sleep and increased sleep latency. These findings suggest that *hmgcr*-associated pathways within different PI neuronal populations may differentially influence sleep–wake regulation. Because statins differ in their pharmacological and physicochemical properties, including lipophilicity and tissue distribution, it remains unclear whether these effects extend across different statin classes.

*Drosophila* has conserved all core components of the mevalonate pathway, including *Hmgcr* (Yi et al., 2006). In contrast to mammals, it does not employ this pathway for *de novo* cholesterol synthesis, owing to the absence of the squalene-producing step, and instead acquires cholesterol entirely from the diet (Niwa and Niwa, 2014; Niwa and Niwa, 2011; Rawson, 2003). This unique feature makes *Drosophila* a valuable pharmacological model for investigating the non-sterol branches of the mevalonate pathway independently of cholesterol biosynthesis. These non-sterol branches regulate several important cellular processes, including isoprenoid-mediated signaling, protein prenylation, N-linked glycosylation, and mitochondrial electron transport-related functions. Disruption of these pathways has been associated with several statin-associated adverse effects and may contribute to alterations in neuronal and behavioral function (Ward et al., 2019; Liao and Laufs, 2005; Laufs and Liao, 2003; German and Liao, 2023). Therefore, *Drosophila* provides a useful system for dissecting the pleiotropic actions of statins, which are often difficult to resolve in clinical studies. Here, we investigated the effects of simvastatin on sleep-wake behavior and sleep architecture in *Drosophila*.

## Result

### Dose- and time-dependent effects of simvastatin on sleep-activity pattern

To precisely quantify sleep–wake behavior in adult wild-type flies, activity was monitored using the *Drosophila* Activity Monitoring (DAM; Trikinetics Inc.) system continuously for five consecutive days under 12:12 h light–dark conditions. Individual flies were housed in DAM system tubes containing food mixed with simvastatin drug at four concentrations (0.0, 0.05, 0.5, and 1.0 mM).

**FIGURE 1:**
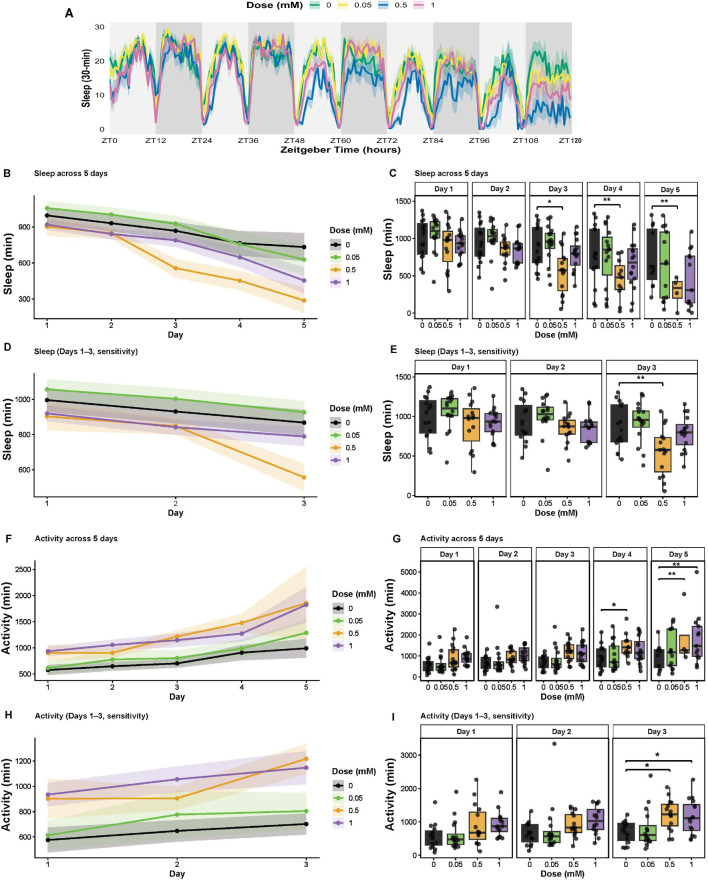
Simvastatin-induced a dose-dependent modulation of sleep-activity patterns in *Drosophila.* **(A)** Sleep profiles for control and each dose concentration are shown over 5 days in a 12:12-h and light:dark cycle. Shaded regions indicate light (ZT 0–12) and dark (ZT 12–24) phases. Treatment groups were 0 mM (initial n = 15), 0.05 mM (n = 16), 0.5 mM (n = 15), and 1 mM (n = 16). By days 5, surviving flies numbered n = 11, 13, 4, and 13, respectively. Lines show mean sleep duration, with shaded bands representing ± SEM. Sleep duration **(B–E)** and locomotor activity **(F–I)** were measured across days 1–5 in flies exposed to increasing doses of simvastatin. The line plots **(B,D,F,H)** show mean ± SEM, and boxplots **(C,E,G,I)** display individual fly data for each dose within each day. Boxes represent the first and third quartiles, center lines indicate medians, whiskers extend to 1.5 x the interquartile range, and points denote individual flies. Data was analyzed using linear mixed models with dose, day, and their interaction as fixed effects and fly ID as a random effect. Post-hoc comparisons on each day were performed using Dunnett’s test comparing each dose to control (0.0 mM), with significance indicated by asterisks (*p < 0.05, **p < 0.01, ***p < 0.001). A sensitivity analysis restricted to days 1–3 **(D,E,H,I)** was performed to assess behavioral changes prior to the onset of mortality, confirms that observed effects are not driven by survival differences. Sample sizes (flies alive) were as follows: days 1–3, n = 15 (0.0 mM), 16 (0.05 mM), 15 (0.5 mM), 16 (1.0 mM); day 4, n = 15 (0.0 mM), 15 (0.05 mM), 11 (0.5 mM), 16 (1.0 mM); day 5, n = 11 (0.0 mM), 13 (0.05 mM), 4 (0.5 mM), 13 (1.0 mM).

Daily sleep duration across Days 1–5 was analyzed using a linear mixed-effects model with dose, day, and their interaction as fixed effects and fly ID as a random effect (Figures 1A–C and Supplementary Table 1). This analysis revealed significant main effects of dose (F (3, 59.6) = 3.81, p = 0.0146) and day (F (4, 207.8) = 48.76, p < 2 × 10^−16^), as well as a significant dose × day interaction (F (12, 207.5) = 1.85, p = 0.042), indicating that the effect of treatment on sleep varied over time. Post hoc comparisons using Dunnett’s correction (with the 0.0 mM group as the reference) identified a significant reduction in sleep at the 0.5 mM dose beginning on Day 3. Flies treated with 0.5 mM simvastatin slept less than controls (556 vs. 868 min; decrease of 312 min, corresponding to a 35.9% reduction, p = 0.013), with the effect becoming more pronounced on Day 4 (418 vs. 765 min; decrease of 347 min, corresponding to a 45.4% reduction, p = 0.007) and Day 5 (236 vs. 684 min; decrease of 447 min, p = 0.005). No significant differences were observed for the 0.05 or 1.0 mM doses at any time point. At the 0.5 mM dose, sleep declined prior to the onset of mortality. Flies began to die on Day 4, with deaths becoming more frequent on Day 5, suggesting that the reduction in sleep is unlikely to be a consequence of mortality, but may instead represent an early effect of treatment. To further confirm that this effect was not influenced by differential survival at later time points, we performed a sensitivity analysis restricted to Days 1–3 (Figures 1D,E and Supplementary Table 2), when all flies were still alive. In this restricted dataset, the linear mixed-effects model again revealed significant effects of dose (F (3, 58) = 3.23, p = 0.029) and day (F (2, 116) = 26.74, p < 2.81 × 10^−10^), as well as a significant dose × day interaction (F (6, 116) = 3.11, p = 0.007). Dunnett-adjusted *post hoc* comparisons showed a significant reduction in sleep in the 0.5 mM group on Day 3 (556 vs. 868 min; decrease of 312 min, a 35.9% reduction, p = 0.002), while no significant differences were observed at earlier time points. These findings suggest that sleep disruption precedes mortality and may occur independently of later time-point survival differences.

Similarly, locomotor activity analyzed over the same 5-day period showed significant main effects of dose (F (3, 60.4) = 4.17, p = 0.009) and day (F (4, 208.8) = 22.29, p < 2.43 × 10^−15^), with no significant dose × day interaction (p = 0.545), indicating that the effect of dose on activity did not vary significantly across days. Post hoc analyses revealed increased locomotor activity in the 0.5 mM group on Day 4 compared with controls (1498 vs. 908 min; increase of 590 min, corresponding to a 65.0% increase, p = 0.033), with a further increase on Day 5 (1951 vs. 1031 min; increase of 920 min, corresponding to an 89.2% increase, p = 0.007). The 1.0 mM group also showed increased activity on Day 5 (1825 vs. 1031 min; increase of 794 min, corresponding to a 77.0% increase, p = 0.003), whereas no significant differences were observed in the 0.05 mM group at any time point (Figures 1F,G and Supplementary Table 3). To further assess whether changes in locomotor activity were influenced by differential survival, we performed sensitivity analyses restricted to Days 1–3, when all flies were alive, using the same linear mixed-effects modeling approach. In this subset, the model revealed significant main effects of dose (F (3, 58) = 3.59, p = 0.019) and day (F (2, 116) = 8.07, p = 0.0005), while the dose × day interaction remained non-significant (p = 0.564). Dunnett-adjusted *post hoc* comparisons showed increased locomotor activity in the 0.5 mM (1217 vs. 703 min; increase of 514 min, corresponding to a 73.1% increase, p = 0.012) and 1.0 mM groups (1147 vs. 703 min; increase of 444 min, corresponding to a 63.2% increase, p = 0.032) compared with controls on Day 3, while no significant differences were observed at earlier time points (Figures 1F,I and Supplementary Table 4).

### Intermediate-dose simvastatin produces marked sleep disruption and reduced survival

**FIGURE 2:**
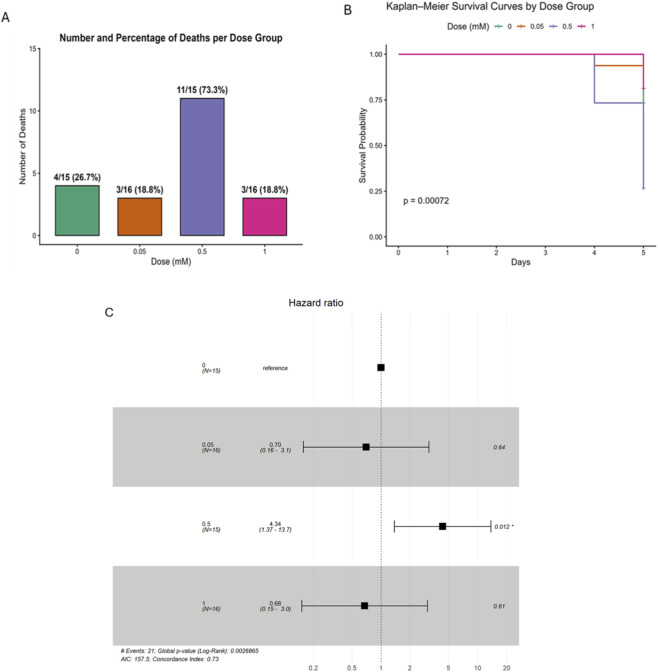
Dose-dependent effects of simvastatin on fly survival. **(A)** Bar plot shows the number and percentage of flies that died during the experimental period for each dose group: 0.0 mM (4/15, 26.7%), 0.05 mM (3/16, 18.8%), 0.5 mM (11/15, 73.3%), and 1.0 mM (3/16, 18.8%). Bars represent absolute counts of deaths, with percentages indicated above each bar. **(B)** Survival probability over time was further evaluated using Kaplan–Meier survival curves. Differences between dose groups and control (0.0 mM) were assessed using the log-rank test. **(C)** Cox proportional hazards regression assessing the effect of dose on survival, with control (0 mM) as the reference group. Sample sizes per group were control (0 mM), n = 15; 0.05, n = 16; 0.5, n = 15; and 1.0, n = 16 flies.

As increased mortality was observed at higher doses during the later days of the experiment, we next examined the effects of simvastatin on fly survival using Kaplan–Meier analysis followed by Cox proportional hazards regression. Kaplan–Meier survival curves revealed a significant overall difference among treatment groups (global log-rank test, p = 0.0007) (Figures 2A,B). Pairwise log-rank tests with Benjamini–Hochberg correction showed that flies exposed to the 0.5 mM dose had reduced survival compared with controls (adjusted p = 0.021). No significant differences were observed between controls and either the 0.05 mM or 1.0 mM groups (adjusted p > 0.05). Consistent with these findings, Cox proportional hazards regression demonstrated a significant overall effect of dose on survival (likelihood ratio test χ^2^ = 14.17, p = 0.003). Relative to controls (0 mM), flies treated with 0.5 mM simvastatin exhibited an increased hazard of death (hazard ratio [HR] = 4.34, 95% CI 1.37–13.70, p = 0.012), corresponding to an approximately 4.3-fold higher mortality risk (Figure 2C). No significant differences in mortality risk were detected for the 0.05 mM (HR = 0.70, p = 0.64) or 1.0 mM (HR = 0.68, p = 0.61) groups. The increased mortality at the 0.5 mM dose coincided with marked sleep loss, suggesting a potential association between sleep disruption and reduced survival.

### Dose-dependent alterations in sleep architecture

**FIGURE 3:**
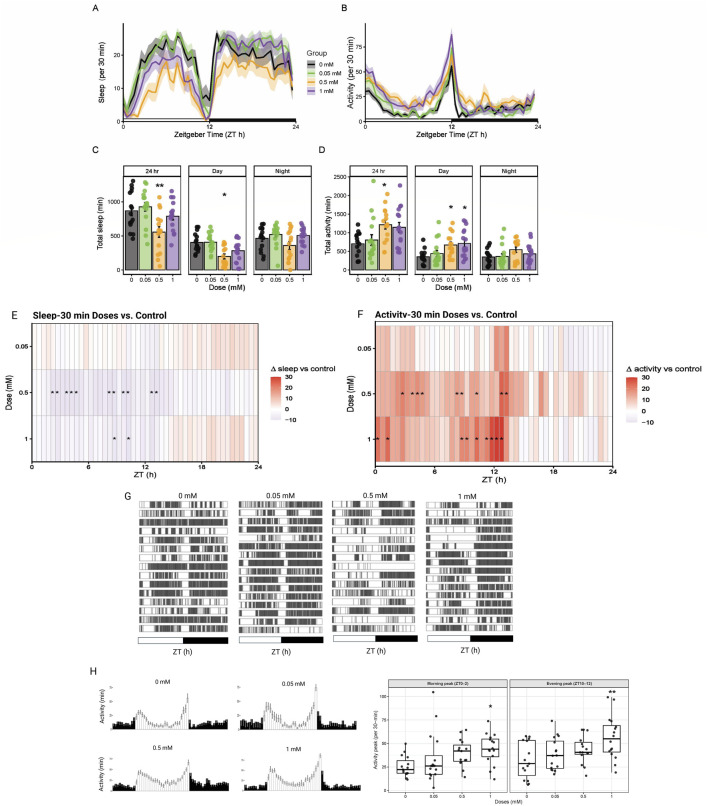
Simvastatin decreased sleep and increased the locomotor activity on the third day and with mainly effect on the midday siesta. **(A,B)** Sleep and activity profiles under 12:12 h, light: dark condition. Mean ± SEM sleep is shown for each dose (0.0, 0.05, 0.5, 1.0 mM). White bars indicate daytime (ZT 0–12); black bars indicate night-time (ZT 12–24). **(C,D)** Total sleep in 24 h, daytime and nighttime. Bars show mean ± SEM. Significant sleep reductions were observed at 0.5 mM during 24 h (p = 0.006) and daytime (p = 0.012). Total locomotor activity in 24 h, daytime and nighttime. Activity significantly increased at 0.5 mM compared to control during 24 h (p = 0.040); during daytime 0.5 mm (p = 0.033) and 1. mM (p = 0.021) differing from control (0.0 mM). (E–F) Sleep difference heatmap (doses vs. control). Red colors indicate increased sleep relative to control. Blue colors indicate reduced sleep (sleep loss). Nighttime (ZT 12–24) is shaded. Asterisks mark ZT bins with significant differences (*p* < 0.05). Red colors indicate higher activity relative to control; blue colors indicate lower activity. **(G)** Raster plots showing the sleep-wake pattern of individual flies treated with simvastatin with various concentration during a 24 h (12 h:12 h dark: light) period. White regions indicate wakefulness - defined as movement or inactivity lasting less than 5 min - whereas black regions denote sleep, characterized by inactivity lasting longer than 5 min. Control flies’ group (0.0 mM), n = 15. Different doses of simvastatin: 0.05 mM, n = 16; 0.5 mM, n = 15; 1.0 mM, n = 16. **(H)** Left: activity pattern for the control group (0.0 mM) and different doses of simvastatin. Right: Morning activity peak was significantly increased at 1 mM (p = 0.011); while Evening activity peak was significantly increased at 1 mM (p = 0.002). Each point represents an individual fly; Boxes represent the first and third quartiles, the center line indicates the median, and whiskers extend to 1.5 x the interquartile range. Statistical significance was assessed using one-way ANOVA with Dunnett’s multiple-comparisons test for normally distributed data, or the Kruskal–Wallis test with Dunn’s multiple-comparisons test when normality was not met. *p < 0.05, **p < 0.001, ***p < 0.0001 compared with control.

Because sleep exhibited a significant dose × day interaction, with a pronounced reduction emerging on Day 3 in the absence of mortality, subsequent analyses focused on this time point to further examine sleep architecture. Simvastatin significantly affected total 24-h sleep duration (one-way ANOVA, p = 0.0018). Post hoc analysis revealed a reduction in sleep exclusively at the 0.5 mM concentration compared with controls (Dunnett’s test, p = 0.006), with mean sleep decreasing from 867.8 min in the control group to 556.4 min in the 0.5 mM group (mean difference = − 311.4 min; corresponding to a 35.9% reduction). The 0.05 and 1.0 mM groups (926.4 min and 788.9 min, respectively) did not differ significantly from controls (Figures 3A,C,G). The effect size for the control versus 0.5 mM comparison was large (Cohen’s d = 1.06, 95% CI 0.29–1.82). To further characterize this effect, a significant treatment effect was observed during the daytime (Kruskal–Wallis test, p = 0.001), driven by reduced sleep at the 0.5 mM dose compared with controls (Dunn’s test, p = 0.012). Mean daytime sleep duration decreased from 403.8 min in controls to 198.7 min in the 0.5 mM group (50.8% reduction). The 0.05 and 1.0 mM groups (406.5 min and 283.2 min, respectively) did not differ significantly from controls. In contrast, no significant effect was observed during the nighttime period (Kruskal–Walli’s test, p = 0.166). Mean sleep values were comparable across groups (control: 464.0 min; 0.05 mM: 519.9 min; 0.5 mM: 357.7 min; 1.0 mM: 505.7 min), and *post hoc* Dunn’s comparisons did not reveal significant differences between any treatment group and controls.

Similarly, total 24-h locomotor activity was significantly altered by simvastatin (Kruskal–Wallis test, p = 0.007). Post hoc Dunn’s analysis revealed increased activity at the 0.5 mM dose compared with controls (p = 0.040), with mean activity increasing from 702.6 min in controls to 1216.9 min in the 0.5 mM group (73.2% increase). The 0.05 and 1.0 mM groups (804.6 min and 1146.9 min, respectively) did not differ significantly from controls (Figures 3B,D). Daytime locomotor activity was also significantly affected by simvastatin (Kruskal–Wallis test, p = 0.0032). Post hoc Dunn’s analysis revealed increased activity at both the 0.5 and 1.0 mM doses compared with controls (p = 0.033 and p = 0.021, respectively), with mean activity rising from 353.3 min in controls to 672.3 min at 0.5 mM (90.3% increase) and 712.9 min at 1.0 mM (101.8% increase). The 0.05 mM group (441.0 min) did not differ significantly from controls. Nighttime locomotor activity was not significantly affected by simvastatin (Kruskal–Wallis test, p = 0.177). Mean activity values were comparable across groups (control: 349.3 min; 0.05 mM: 363.6 min; 0.5 mM: 544.6 min; 1.0 mM: 434.0 min), and Dunn’s *post hoc* analysis did not reveal significant differences between any treatment group and controls.

### Dose-dependent reductions in midday siesta sleep are accompanied by increased locomotor activity

A more detailed time-resolved analysis revealed that simvastatin produced dose-dependent reductions in sleep duration and concomitant increases in locomotor activity from the early daytime period through the midday siesta (ZT 3–9), a phase typically characterized by elevated sleep and reduced activity in control flies, with effects extending into the early night. At the 0.5 mM dose, sleep reductions were evident from the early daytime period and became more pronounced during the midday siesta (ZT 3–9). These reductions were consistent across multiple time points and extended into the late day and early night. In contrast, the 1.0 mM dose produced more limited effects (Figure 3E). Detailed ZT-specific comparisons are provided in Supplementary Table 5. Consistent with these sleep effects, locomotor activity increased in a dose-dependent manner (Figure 3F). Activity was elevated at 1.0 mM during the early morning (ZT 0–2), whereas activity increases during the midday siesta were more pronounced at 0.5 mM. Elevated activity persisted into the late day and early night, particularly at higher doses. Detailed ZT-resolved data are provided in Supplementary Table 6.

Peak-based analyses supported these findings. Morning activity (M peak; ZT 0–2) differed significantly across groups (Kruskal–Wallis test, p = 0.005), with increased activity at the 1.0 mM dose compared with controls (25.6 vs. 44.2; +18.6, p = 0.011), while no significant effects were observed at 0.5 or 0.05 mM. Evening activity (E peak; ZT 10–12) also differed significantly across groups (one-way ANOVA, p = 0.006), with increased activity at the 1.0 mM dose (32.1 vs. 56.2; +24.1, p = 0.002), whereas other doses showed no significant changes (Figure 3H).

### Simvastatin disrupts sleep–wake episode architecture

**FIGURE 4:**
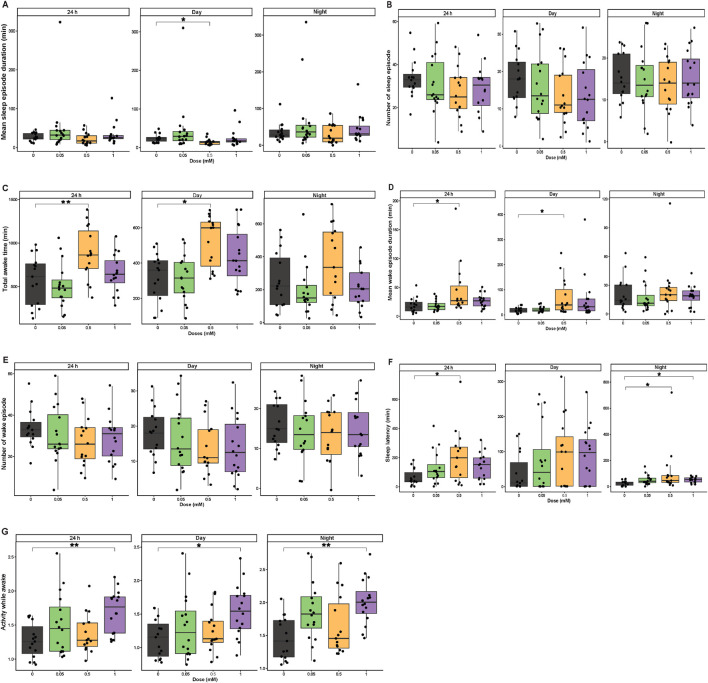
Simvastatin disrupts sleep parameters. **(A–G)** Mean sleep episode duration, number of sleep episode, total awake duration, mean wake episode duration, number of wake episode, sleep latency and activity while awake for control (0.0 mM, n = 15) and different simvastatin concentrations (0.05 mM, n = 16; 0.5 mM, n = 15; 1.0 mM, n = 16). Boxes represent the first and third quartiles, center lines indicate medians, whiskers extend to 1.5 x the interquartile range, and points denote individual flies. It was performed One-way ANOVA to detect the significant of different simvastatin concentrations effects compared to control, for daytime mean sleep episode duration (P = 0.041), total awake time duration (P = 0.006), daytime awake time duration (P = 0.012), total mean wake episode duration (P = 0.047), daytime mean wake episode duration (P = 0.044), total sleep latency (P = 0.028), nighttime sleep latency for 0.5 mM vs. control (P = 0.021) and 1.0 mM vs. control (P = 0.028). Total activity while awake for 1.0 mM vs. control (P = 0.005); daytime activity while awake for 1.0 mM vs. control (P = 0.011), nighttime activity while awake for 1.0 mM vs. control (P = 0.001).

To further understand the reduction in total sleep duration at 0.5 mM, we examined changes in sleep architecture. The reduction in total sleep duration was primarily driven by decreased daytime sleep, attributable to shorter sleep episode duration and longer wake bouts during the day and was accompanied by increased sleep latency at night. Furthermore, total 24-h sleep episode duration did not differ significantly between groups. In contrast, daytime sleep episode duration differed significantly (Kruskal–Wallis test, p = 0.0096), with a reduction at the 0.5 mM dose (24.3 vs. 14.6 min; − 9.7 min, p = 0.041), while no differences were observed at other doses. Nighttime sleep episode duration was also not significantly affected (Figure 4A). These findings suggest that the 0.5 mM dose selectively reduces daytime sleep episode duration, consistent with increased sleep fragmentation. Notably, the total number of sleep episodes did not differ between groups over 24 h, and no differences were observed during either the daytime or nighttime (Figure 4B). These findings are consistent with alterations in sleep structure rather than changes in overall sleep quantity, suggesting potential modulation of sleep homeostasis. Under normal conditions, a reduction in sleep episode duration would be expected to increase sleep pressure and promote compensatory increases in episode number. However, despite reduced daytime sleep episode duration, no such increase was observed.

Total 24-h wake time increased at 0.5 mM (572 vs. 884 min; +312 min, p = 0.006), with a similar increase during the daytime (316 vs. 521 min; +205 min, p = 0.012). Mean wake episode duration was also increased at 0.5 mM over 24 h (19.1 vs. 45.5 min; +26.4 min, p = 0.047) and during the daytime (18.7 vs. 69.3 min; +50.6 min, p = 0.044) (Figures 4C,D). The number of wake episodes remained unchanged across groups over 24 h as well as during daytime and nighttime periods (Figure 4E).

### Simvastatin prolongs sleep latency and enhances wake activity at higher concentrations

Sleep latency over 24 h differed significantly between groups, with an increase at the 0.5 mM dose (64.7 vs. 205 min; +140.3 min, corresponding to a 216.8% increase, p = 0.028). In the nighttime sleep latency differed significantly, with increases at both 0.5 mM (25.1 vs. 111 min; + 85.9 min, corresponding to a 342.2% increase, p = 0.021) and 1.0 mM doses (25.1 vs. 52.3 min; + 27.2 min, corresponding to a 108.4% increase, p = 0.028) (Figure 4F). In addition, across 24 h, waking activity intensity differed significantly between groups, driven by an increase at the 1.0 mM dose (1.27 vs. 1.71; +0.44, corresponding to a 34.6% increase, p = 0.005). During the daytime, activity intensity differed significantly across groups. Activity intensity increased at 1.0 mM (1.14 vs. 1.55; +0.41, corresponding to a 36.0% increase, p = 0.011). A similar pattern was observed at night, with higher intensity at 1.0 mM (1.46 vs. 2.02; + 0.56, corresponding to a 38.4% increase, p = 0.001) (Figure 4G). These findings suggest that the increased waking activity intensity may reflect heightened arousal. Importantly, waking activity at the 0.5 mM dose was unchanged compared with control (Figure 4G), suggesting that the reduction in sleep is unlikely to be attributable to generalized hyperactivity. Notably, nighttime sleep, sleep episode duration, and episode number were unchanged, whereas sleep latency was prolonged. This pattern suggests a possible impairment in sleep initiation despite preserved overall sleep at night. Together, these findings may indicate that impaired sleep initiation limits the expression of compensatory homeostatic responses.

### Simvastatin modulates sleep pressure and depth

Sleep pressure and sleep depth are central regulators of wake and sleep, reflecting internal sleep drive and sleep quality, respectively. Because traditional methods for quantifying these parameters in *Drosophila* are constrained by low temporal resolution and often require sleep disruption, we used a high-resolution, noninvasive DAM-based approach, following established methods (Wiggin et al., 2020), to quantify the probability of transitioning from wake to sleep P (doze) as a measure of sleep pressure and from sleep to wake P (wake) as a measure of sleep depth, thereby assessing the effects of simvastatin on sleep state dynamics.

**FIGURE 5:**
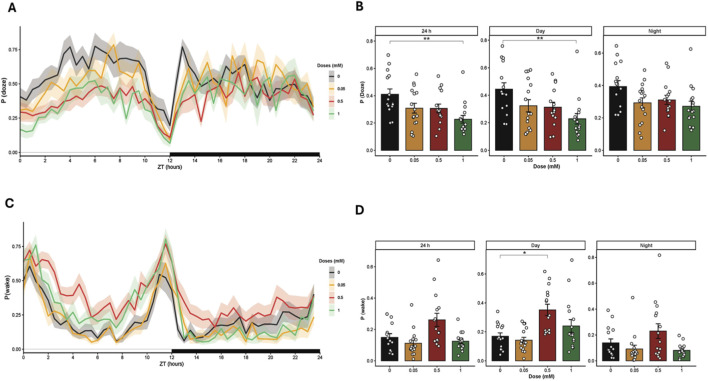
Effects of simvastatin on sleep–wake transition probabilities in *Drosophila*, reflected by changes in P(Doze) and P(Wake). **(A,C)** P (Doze) and P(Wake) profiles were measured under a 12:12 h Light:dark cycle. Data are presented as mean ± SEM for control flies (0.0 mM, n = 15) and drug-treated groups (0.05 mM, n = 16; 0.5 mM, n = 15; 1.0 mM, n = 16). White bars indicate daytime (ZT 0–12), and black bars indicate night-time (ZT 12–24). **(B)** P (Doze) across 24 h was significantly reduced at 1.0 mM compared with control (Kruskal–Wallis test, p = 0.004, Dunn’s test p = 0.001). Daytime was also reduced at 1.0 mM (Kruskal–Wallis test, p = 0.006, daytime: p = 0.002). **(D)** Daytime P(Wake) was significantly increased at 0.5 mM compared with control (Dunn’s test, p = 0.012 after Kruskal–Wallis test, p = 0.001). Data points represent individual flies; bars indicate mean ± SEM.

Across 24 h, P (Doze) differed significantly between groups (Kruskal–Wallis test, p = 0.004). Control flies exhibited a mean value of 0.410, while the 1.0 mM dose reduced P (Doze) to 0.227 (- 0.183, corresponding to a 44.6% reduction, p = 0.001). No significant differences were observed at 0.05 mM or 0.5 mM. During the daytime, P (Doze) also differed significantly (Kruskal–Wallis test, p = 0.006), with a reduction at 1.0 mM (0.444 vs. 0.228; −0.216, corresponding to a 48.6% reduction, p = 0.002), while other doses showed no significant effects. In contrast, nighttime P (Doze) did not differ between groups (Kruskal–Wallis test, p = 0.068), although values were lower at 1.0 mM (0.272 vs. 0.393), with a borderline *post hoc* effect (p = 0.052) (Figures 5A,B).

On the other hand, P(Wake) differed significantly between groups over 24 h (Kruskal–Wallis test, p = 0.005), although no significant pairwise differences were detected (all p > 0.5). During the daytime, P(Wake) also differed significantly (Kruskal–Wallis test, p = 0.001), driven by an increase at the 0.5 mM dose (0.170 vs. 0.352; +0.182, corresponding to a 107.1% increase, p = 0.012), while no effects were observed at other doses. Nighttime P(Wake) did not differ between groups (Figures 5C,D).

Consistent with these findings, sleep–wake transition probabilities showed dose-dependent changes. At 1.0 mM, P (Doze) was reduced across 24 h and during the daytime, suggesting a decreased propensity to initiate sleep. In contrast, at 0.5 mM, P (wake) increased during the daytime, indicating a higher probability of transitioning from sleep to wakefulness. Together, these observations are consistent with dose-specific alterations in sleep–wake dynamics, which may reflect elevated arousal at 0.5 mM and a decreased propensity to initiate sleep at 1.0 mM, and may contribute to the observed changes in sleep architecture across doses.

## Discussion

In this study, we show that simvastatin induces dose-dependent alterations in sleep–wake behavior, sleep architecture, and survival in *Drosophila*. Notably, the intermediate dose (0.5 mM) was associated with a reduction in total sleep duration, primarily driven by decreased daytime sleep, accompanied by shorter sleep episode duration and increased sleep latency. In contrast, the higher dose (1.0 mM) exhibited a distinct pattern, with preserved total sleep duration but altered sleep architecture, characterized by increased waking activity intensity and prolonged sleep latency. High-resolution analysis of sleep–wake transitions further revealed dose-specific changes, including increased P(Wake) during the daytime at 0.5 mM and reduced P (Doze) at 1.0 mM across 24 h and during the daytime, indicating distinct alterations in sleep–wake transition dynamics. Sleep is regulated by a dynamic balance between sleep-promoting and arousal-related processes (Zielinski et al., 2016). The present findings suggest that simvastatin may be associated with alterations in this balance, reflected by changes in sleep–wake behavior and transition dynamics. Together, these results indicate that simvastatin may influence multiple components of sleep–wake regulation, including sleep initiation, stability, and overall architecture.

At 0.5 mM, the reduction in total sleep appears to be largely driven by decreased daytime (siesta) sleep, while nighttime sleep remains relatively preserved, suggesting that the effect may not be uniform across the 24-h cycle. Sleep episode number is not significantly altered, which may indicate reduced consolidation without clear compensatory increases in episode frequency. In addition, sleep latency is increased during the night but not during the day, suggesting a potential selective effect on sleep initiation. Notably, waking activity at this dose is not increased, which may indicate that the reduction in sleep is not primarily driven by generalized hyperactivity or heightened arousal. Consistent with previous work, these findings are compatible with the interpretation that sleep onset and sleep maintenance are regulated by partially distinct mechanisms (), both of which may be affected under these conditions, potentially contributing to an overall disruption of sleep–wake regulation.

At 1.0 mM, total sleep time remains largely preserved despite increased waking activity and delayed sleep onset, suggesting a qualitatively distinct alteration in sleep architecture. The increase in waking activity may reflect a shift toward elevated arousal or wake drive, although this interpretation remains indirect. Alternatively, the increased locomotor activity observed at 1.0 mM simvastatin may have contributed to fatigue, which in turn may have contributed to the relative preservation of total sleep duration. Consistent with this possibility, previous studies have reported that statin exposure in *Drosophila* is associated with muscular and locomotor alterations (Al-Sabri et al., 2022). Therefore, the preserved total sleep duration observed at 1.0 mM simvastatin may partly reflect fatigue-related responses secondary to prolonged wake activity and statin-associated muscular effects. The drug may therefore influence sleep initiation and alter sleep architecture without substantially changing total sleep duration, a dissociation that has been reported in *Drosophila* (Liu et al., 2019). In this context, the dissociation between preserved sleep quantity and altered sleep initiation and wake activity may also be consistent with partially distinct regulation of sleep initiation and maintenance, suggesting that simvastatin may affect specific components of sleep–wake control rather than uniformly reducing sleep. Taken together, these findings suggest that the phenotype may reflect a combination of altered sleep initiation, reduced sleep stability, and dose-dependent changes in arousal, with a notable effect on daytime sleep regulation. This pattern may be more consistent with a redistribution of sleep across the circadian cycle than with a uniform reduction in sleep across the 24-h period.

The survival analyses indicate a non-linear dose–response relationship, with increased mortality observed selectively at the intermediate simvastatin concentration. This pattern suggests that lethality may not be a simple function of cumulative drug exposure. Non-monotonic dose–response relationships are well described in biological systems, where different concentrations can produce distinct or even opposing physiological effects (Calabrese and Baldwin, 2002a; Vandenberg et al., 2012), and such responses have been reported across a range of neurobehavioral and pharmacological contexts (Calabrese, 2008; Calabrese and Baldwin, 2002b). In the present study, the intermediate dose (0.5 mM) was associated with both increased mortality and more pronounced alterations in sleep–wake behavior, whereas the higher dose (1.0 mM) preserved total sleep but was associated with increased sleep latency and waking activity. This pattern suggests a dose-dependent but qualitatively distinct behavioral response, rather than a simple monotonic effect. Importantly, the behavioral changes were observed prior to the onset of overt mortality, indicating that they are unlikely to be solely attributable to late-stage physiological decline. At the same time, it cannot be excluded that some aspects of the observed phenotype reflect broader physiological effects of drug exposure, including potential sublethal toxicity. The temporal association between altered sleep–wake behavior and increased mortality raises the possibility that changes in behavioral or physiological state may contribute to reduced survival; however, this relationship remains correlative and should be interpreted with caution. Notably, the presence of dose-specific and qualitatively distinct behavioral patterns, together with the absence of a uniform reduction in locomotor activity, may be less consistent with a generalized, non-specific impairment, although this does not exclude contributions from toxicity.

The non-linear dose-response relationship observed in the present study also suggests that concentration-dependent differences in target selectivity and downstream pathway interactions may contribute to the observed behavioral phenotypes. At lower or intermediate concentrations, simvastatin may preferentially affect primary high-affinity targets associated with Hmgcr-related pathways, whereas higher concentrations could additionally influence secondary or off-target pathways as primary targets become increasingly saturated. Importantly, such concentration-dependent interactions may not necessarily produce progressively greater toxicity. In some cases, additional pathway interactions at higher concentrations could potentially exacerbate adverse effects, whereas in other cases they may partially counteract pathways contributing to behavioral alterations observed at lower concentrations. These possibilities may partly contribute to the distinct phenotypes observed between the 0.5 mM and 1.0 mM simvastatin treatment groups. However, because the specific downstream molecular pathways involved were not directly examined in the present study, these mechanisms remain speculative and warrant further investigation. Overall, the underlying mechanisms linking simvastatin exposure, behavioral alterations, and survival cannot be determined from the current data and will require further investigation.

Because simvastatin was administered through standard fly food, differences in feeding behavior may have influenced actual drug exposure. We did not directly measure food intake in the present study; therefore, we cannot exclude the possibility that simvastatin altered feeding or that different doses resulted in different levels of drug consumption. This is particularly relevant because gastrointestinal side effects are common in patients treated with simvastatin, and emesis-like responses have been described in *Drosophila* (Shi et al., 2025). In addition, previous work showed that 0.5 mM fluvastatin, another lipophilic statin, increased food intake in *Drosophila* through altered insulin-related signaling (Williams et al., 2022). It is therefore possible that 0.5 mM simvastatin could have produced similar changes in feeding, potentially increasing drug exposure and contributing to the stronger behavioral and survival phenotype observed at this dose. Conversely, reduced consumption of food containing 1.0 mM simvastatin could potentially occur as a result of aversive gastrointestinal or emesis-like responses, hypothetically leading to lower effective internal exposure and reduced hemolymph drug concentrations. This may partly explain the preserved total sleep duration and lower mortality observed at this concentration. However, food intake and hemolymph simvastatin levels were not measured, and the basis for the distinct phenotype at 1.0 mM remains unclear. Future studies should directly quantify feeding behavior and internal drug exposure to determine whether the observed effects were due to simvastatin concentration itself or differences in food consumption between treatment groups.

Prior genetic studies have implicated Hmgcr in the regulation of sleep-related processes, raising the possibility that simvastatin may influence sleep–wake behavior through related pathways. However, the underlying mechanisms were not directly examined in the present study. *Drosophila* lacks the capacity for *de novo* cholesterol synthesis and depends on dietary sterols (Niwa and Niwa, 2014; Niwa and Niwa, 2011; Rawson, 2003), providing a system in which inhibition of Hmgcr is expected to predominantly affect cholesterol-independent branches of the mevalonate pathway. Accordingly, the observed phenotypes may be consistent with alterations in isoprenoid-dependent signaling processes, although this remains speculative and requires further investigation. Supporting this possibility, previous work has shown that targeted knockdown of hmgcr in DH44-expressing neurons is associated with reduced sleep and prolonged sleep latency, producing a phenotype that resembles aspects of the effects observed here (Alsehli et al., 2022). DH44 neurons, the *Drosophila* homolog of corticotropin-releasing factor-related signaling neurons, have been implicated in the regulation of arousal, stress-related responses, feeding behavior, and sleep–wake regulation (Dus et al., 2015; Cavanaugh et al., 2014; Cannell et al., 2016; Poe et al., 2024).

Importantly, because *Drosophila* lacks the squalene-producing step required for *de novo* cholesterol synthesis, this model may be particularly useful for examining pleiotropic statin effects in isolation from cholesterol biosynthesis. In addition, disruption of small GTPase signaling in *Drosophila*, which depends on isoprenoid-mediated prenylation, has been linked to alterations in sleep homeostasis and cognitive functions (Donlea et al., 2014). Other non-sterol branches of the mevalonate pathway, including N-linked glycosylation and mitochondrial electron transport-related processes, may also contribute to the behavioral effects associated with statin exposure. Together, these findings suggest that pathways downstream of *hmgcr* may contribute to sleep–wake regulation, although their relevance to the present observations remains to be established. Overall, these findings identify measurable effects of simvastatin on sleep and activity in *Drosophila* and suggest that this model may be useful for investigating the potential neuromodulatory consequences of statin exposure, although further studies will be required to validate and extend these observations.

Several limitations of this study should be considered when interpreting the findings. First, although the observed behavioral changes are consistent with alterations in sleep regulation, it cannot be excluded that some of the effects may be driven by dose-dependent toxicity, particularly at intermediate concentration. This is especially relevant given that the dose producing the most pronounced behavioral phenotype also showed reduced survival. While we attempted to address this by analyzing early time points prior to mortality, however; this approach may not be sufficient to fully exclude non-specific physiological impairment or sublethal toxicity effects. The persistence of the phenotype at these stages suggests that the observed effects are not solely attributable to late-stage toxicity or pre-mortem changes; however, the relative contribution of toxicity versus specific effects on sleep homeostasis cannot be fully resolved within the current study. In addition, more subtle physiological influences, including potential alterations in feeding behavior, metabolic state, or general vitality, cannot be excluded and may contribute to the observed changes in sleep and activity. We did not directly assess food intake, and it remains possible that the presence of simvastatin in the medium may have influenced feeding behavior, which could in turn affect sleep and locomotor activity. Second, only male flies aged 3–7 days were used. While this approach employs fully developed adult flies and reduces variability associated with sex-specific differences, including reproductive and behavioral variation, it may limit the generalizability of the findings to other populations. In addition, the relatively broad age range may introduce variability in behavioral responses, as even within this window, subtle differences in sleep and locomotor activity may occur. Furthermore, the present study evaluated simvastatin exposure over a relatively short treatment period in young adult flies; therefore, the findings may not fully reflect the effects of chronic exposure or responses in aged animals. Previous studies have also implicated insulin signaling pathways in *Drosophila* statin responses (Williams et al., 2022), and because neuronal and metabolic signaling pathways may differ with age, behavioral responses to simvastatin could vary in older flies. In addition, the effects of long-term survival and chronic simvastatin exposure were not assessed in the present study.

Third, the study relies on a single methodological approach, namely, DAM system-based activity and sleep monitoring. While this method is widely used and well established for assessing sleep and locomotor activity in *Drosophila*, the use of complementary experimental approaches would further strengthen the conclusions. In addition, the study was conducted as a single experimental cohort and has not been independently replicated. Although multiple individuals per group were analyzed using a longitudinal design, independent replication will be necessary to confirm the reproducibility and generalizability of the observed behavioral and mortality effects. Fourth, the number of flies analyzed per group is relatively modest, although it is within the range commonly used in *Drosophila* behavioral studies. Nevertheless, larger sample sizes would increase the statistical power and robustness of the findings. Fifth, all flies were housed individually in DAM tubes for five consecutive days. Although single-fly housing is standard for DAM-based sleep analysis and was applied equally across control and treatment groups, social isolation may influence baseline sleep and locomotor behavior and could potentially modify the response to simvastatin. While this does not affect internal comparisons, it may influence the interpretation of baseline conditions and the generalizability of the findings. Finally, further studies will be required to validate these findings, clarify the underlying mechanisms, and disentangle the relative contributions of toxicity, altered physiological state, and sleep-related effects.

## Methods

### Fly stock and maintenance

Flies were reared on standard fly food (Jazz mix, Fisher Scientific, Gothenburg, Sweden) supplemented with yeast extract (VWR, Stockholm, Sweden). Adult male flies aged 3–7 days were used for the experiment to minimize variability associated with sex-specific differences and age-dependent changes in behavior and to ensure stable sleep patterns in fully developed adult flies. Flies were maintained at 25 °C in an incubator at 60% humidity under a 12:12 h light:dark cycle. The CSORC lab strain was generated by crossing the wild-type strains Canton-S and Oregon-R-C, both originally obtained from the Bloomington *Drosophila* Stock Center (Bloomington, IN, USA) and maintained in our laboratory for several years. Oregon-R-C corresponds to BDSC stock #5, while Canton-S corresponds to BDSC stock #64349.

### Locomotor activity

Locomotor activity was recorded using the *Drosophila* Activity Monitor (DAM) system (Trikinetics, Waltham).) under 12:12 h light: dark conditions. Flies were introduced into DAM monitoring tubes at 14:00 (ZT6), and data collection was initiated the following day at 08:00 (ZT0), allowing an acclimation period of approximately 18 h prior to analysis. Behavioral data were then recorded continuously for five consecutive days in 24-h cycles (ZT0–ZT24). DAM output files were imported into MATLAB, where sleep and locomotor activity were analyzed using a signal-processing toolbox implemented in MATLAB. Sleep was defined as periods of inactivity lasting longer than 5 min (Hendricks et al., 2000; Shaw et al., 2000). Sleep latency was defined as the interval from lights-off to the onset of the first sleep episode.

Sleep–wake transition probabilities were calculated using 1-min activity bins (Wiggin et al., 2020). P (wake) was defined as the probability of transitioning from inactivity to activity and was calculated as

$$P\left(\text{wake}\right)=\frac{N_{\text{inactive}\to \text{active}}}{N_{\text{inactive}}}$$

where $N_{\text{inactive}}$ represents the total number of inactive bins, excluding the final inactive bin, and $N_{\text{inactive}\to \text{active}}$ represents the number of inactive bins followed by activity in the subsequent bin. If a fly never entered an inactive state, P (wake) was considered undefined.

P (doze) was calculated analogously as the probability of transitioning from activity to inactivity,

$$P\left(\text{doze}\right)=\frac{N_{\text{active}\to \text{inactive}}}{N_{\text{active}}}$$

where $N_{\text{active}}$ represents the total number of active bins, excluding the final active bin.

### Simvastatin experiment

Simvastatin (Sigma-Aldrich, S6196) was dissolved in ethanol to prepare a 100 mM stock solution. The stock was diluted into 5 mL of standard fly lab food to final concentrations of 0.0, 0.05, 0.5, and 1 mM. The final ethanol concentration was maintained at 1% in all treatment and control groups.

## Data analysis

The experiments and subsequent data processing were not blinded. Flies were randomly allocated to control and treatment groups. Statistical analyses were performed using R (version 4.5.1). Sleep duration and locomotor activity were analyzed using linear mixed-effects models to account for repeated measurements from individual flies across multiple days. Dose, day, and their interaction were included as fixed effects, with fly identity included as a random effect. Post hoc comparisons against the control group were performed using Dunnett’s test, with p values adjusted for multiple comparisons.

For single-time-window comparisons, data distribution was assessed using the Shapiro–Wilk normality test. Normally distributed data were analyzed using one-way analysis of variance (ANOVA), followed by Dunnett’s *post hoc* test for comparisons with the control group. When normality assumptions were violated, non-parametric Kruskal–Wallis tests were used, followed by Dunn’s *post hoc* test with Bonferroni correction. All values are reported as mean behavioral responses with the standard error of the mean (SEM). Statistical significance was defined as p < 0.05.

## Data Availability

The original contributions presented in the study are included in the article/Supplementary Material, further inquiries can be directed to the corresponding author.
