# Supplementary material for: Simvastatin modulates sleep–wake behavior and locomotor activity in a dose-dependent manner in Drosophila
Source: Front Pharmacol. 2026 Jul 1;17:1815042. doi: 10.3389/fphar.2026.1815042 (PMC13369620; doi:10.3389/fphar.2026.1815042)
Supplement: Supplementary file 1 [file Table1.docx]

**Table 1. Effect of simvastatin on sleep duration across five consecutive days in wild type flies.**

Daily sleep duration (minutes) of flies treated with different doses of simvastatin (0, 0.05, 0.5, and 1.0 mM) over five consecutive days. Values represent estimated marginal means (95% confidence intervals) derived from a linear mixed-effects model with dose, day, and their interaction as fixed effects and fly ID as a random effect. Changes relative to the control group (0 mM) are expressed as increase or decrease in minutes. P-values correspond to pairwise comparisons versus control using Dunnett’s correction. N indicates the number of flies contributing data at each time point; reduced sample size at later days reflects mortality.

| Day | Dose (mM) | N | Mean sleep (min) (95% CI) | Change vs control (min) | p-value |
| --- | --- | --- | --- | --- | --- |
| 1 | Control (Reference) | 15 | 996 (845–1147) | Control (Reference) | NA |
| 1 | 0.05 | 16 | 1057 (910–1203) | + 61 min | 0.863 |
| 1 | 0.5 | 15 | 903 (752–1054) | - 93 min | 0.703 |
| 1 | 1 | 16 | 920 (774–1067) | - 76 min | 0.79 |
| 2 | Control (Reference) | 15 | 931 (780–1082) | Control (Reference) | NA |
| 2 | 0.05 | 16 | 1003 (857–1150) | + 72 min | 0.807 |
| 2 | 0.5 | 15 | 849 (697–1000) | - 82 min | 0.762 |
| 2 | 1 | 16 | 842 (695–988) | - 89 min | 0.715 |
| 3 | Control (Reference) | 15 | 868 (717–1019) | Control (Reference) | NA |
| 3 | 0.05 | 16 | 926 (780–1073) | + 58 min | 0.873 |
| 3 | 0.5 | 15 | **556 (405–708)** | **- 312 min** | **0.013** |
| 3 | 1 | 16 | 789 (642–935) | - 79 min | 0.773 |
| 4 | Control (Reference) | 15 | 765 (614–916) | Control (Reference) | NA |
| 4 | 0.05 | 15 | 750 (601–899) | - 15 min | 0.993 |
| 4 | 0.5 | 11 | **418 (254–582)** | **- 347 min** | **0.007** |
| 4 | 1 | 16 | 648 (501–794) | - 117 min | 0.547 |
| 5 | Control (Reference) | 11 | 684 (520–847) | Control (Reference) | NA |
| 5 | 0.05 | 13 | 584 (430–738) | - 100 min | 0.691 |
| 5 | 0.5 | 4 | **236 (8–464)** | **- 448 min** | **0.005** |
| 5 | 1 | 13 | 443 (289–597) | - 241 min | 0.094 |

Bold values indicate statistically significant differences (p < 0.05).

**Table 2. Effect of simvastatin on sleep duration across Days 1–3 (sensitivity analysis) in wild type flies.**

Daily sleep duration (minutes) across Days 1–3 (sensitivity analysis). Values represent estimated marginal means (95% confidence intervals) from a linear mixed-effects model. Differences are relative to control (0 mM) using Dunnett’s correction. n indicates the number of flies contributing data at each time point.

| Day | Dose (mM) | N | Mean sleep (min) (95% CI) | Change vs control (min) | p-value |
| --- | --- | --- | --- | --- | --- |
| 1 | Control (Reference) | 15 | 996 (845–1147) | Control (Reference) | NA |
| 1 | 0.05 | 16 | 1057 (910–1203) | + 61 min | 0.801 |
| 1 | 0.5 | 15 | 903 (752–1054) | - 93 min | 0.588 |
| 1 | 1 | 16 | 920 (774–1067) | - 76 min | 0.701 |
| 2 | Control (Reference) | 15 | 931 (780–1082) | Control (Reference) | NA |
| 2 | 0.05 | 16 | 1003 (857–1150) | + 72 min | 0.724 |
| 2 | 0.5 | 15 | 849 (697–1000) | - 82 min | 0.663 |
| 2 | 1 | 16 | 842 (695–988) | - 89 min | 0.603 |
| 3 | Control (Reference) | 15 | 868 (717–1019) | Control (Reference) | NA |
| 3 | 0.05 | 16 | 926 (780–1073) | + 58 min | 0.815 |
| 3 | 0.5 | 15 | **556 (405–708)** | **- 312 min** | **0.002** |
| 3 | 1 | 16 | 789 (642–935) | - 79 min | 0.678 |

Bold values indicate statistically significant differences (p < 0.05).

**Table 3. Effect of simvastatin on locomotor activity across five consecutive days in wild type flies**

Daily locomotor activity (minutes) of flies treated with simvastatin (0, 0.05, 0.5, and 1.0 mM) over five consecutive days. Values represent estimated marginal means (95% confidence intervals) derived from a linear mixed-effects model with dose, day, and their interaction as fixed effects and fly ID as a random effect. Changes are expressed relative to the control group (0 mM), and p-values correspond to Dunnett-adjusted comparisons versus control. N indicates the number of flies contributing data at each time point; reduced sample size at later days reflects mortality.

| Day | Dose (mM) | N | Mean activity (min) (95% CI) | Change vs control | p-value |
| --- | --- | --- | --- | --- | --- |
| 1 | Control (Reference) | 15 | 576 (267–885) | Control (Reference) | NA |
| 1 | 0.05 | 16 | 613 (314–912) | + 37 min | 0.989 |
| 1 | 0.5 | 15 | 902 (593–1211) | + 326 min | 0.328 |
| 1 | 1 | 16 | 935 (635–1234) | + 359 min | 0.245 |
| 2 | Control (Reference) | 15 | 648 (339–957) | Control (Reference) | NA |
| 2 | 0.05 | 16 | 776 (477–1075) | + 128 min | 0.854 |
| 2 | 0.5 | 15 | 905 (596–1214) | + 257 min | 0.509 |
| 2 | 1 | 16 | 1055 (756–1354) | + 407 min | 0.159 |
| 3 | Control (Reference) | 15 | 703 (394–1012) | Control (Reference) | NA |
| 3 | 0.05 | 16 | 805 (505–1104) | + 102 min | 0.908 |
| 3 | 0.5 | 15 | 1217 (908–1526) | + 514 min | 0.058 |
| 3 | 1 | 16 | 1147 (848–1446) | + 444 min | 0.112 |
| 4 | Control (Reference) | 15 | 908 (599–1217) | Control (Reference) | NA |
| 4 | 0.05 | 15 | 978 (673–1283) | + 70 min | 0.958 |
| 4 | 0.5 | 11 | **1498 (1159–1838)** | **+ 590 min** | **0.033** |
| 4 | 1 | 16 | 1273 (974–1572) | + 365 min | 0.231 |
| 5 | Control (Reference) | 11 | 1031 (692–1369) | Control (Reference) | NA |
| 5 | 0.05 | 13 | 1351 (1034–1669) | + 320 min | 0.386 |
| 5 | 0.5 | 4 | **1951 (1460–2441)** | **+ 920 min** | **0.007** |
| 5 | 1 | 13 | **1825 (1507–2142)** | **+ 794 min** | **0.003** |

Bold values indicate statistically significant differences (p < 0.05).

**Table 4: Effect of simvastatin on locomotor activity across Days 1–3 (sensitivity analysis) in wild type flies.**

Daily locomotor activity (minutes) across Days 1–3 (sensitivity analysis). Values represent estimated marginal means (95% confidence intervals) from a linear mixed-effects model. Differences are relative to control (0 mM) using Dunnett’s correction. N indicates the number of flies contributing data at each time point.

| Day | Dose (mM) | N | Mean activity (min) (95% CI) | Change vs control | p-value |
| --- | --- | --- | --- | --- | --- |
| 1 | Control (Reference) | 15 | 576 (267–885) | Control (Reference) | NA |
| 1 | 0.05 | 16 | 613 (314–912) | + 37 min | 0.982 |
| 1 | 0.5 | 15 | 902 (593–1211) | + 326 min | 0.166 |
| 1 | 1 | 16 | 935 (635–1234) | + 359 min | 0.105 |
| 2 | Control (Reference) | 15 | 648 (339–957) | Control (Reference) | NA |
| 2 | 0.05 | 16 | 776 (477–1075) | + 128 min | 0.772 |
| 2 | 0.5 | 15 | 905 (596–1214) | + 257 min | 0.332 |
| 2 | 1 | 16 | 1055 (756–1354) | + 407 min | 0.054 |
| 3 | Control (Reference) | 15 | 703 (394–1012) | Control (Reference) | NA |
| 3 | 0.05 | 16 | 805 (505–1104) | + 102 min | 0.854 |
| 3 | 0.5 | 15 | **1217 (908–1526)** | **+ 514 min** | **0.012** |
| 3 | 1 | 16 | **1147 (848–1446)** | **+ 444 min** | **0.032** |

Bold values indicate statistically significant differences (p < 0.05).

**Table 5. Effects of simvastatin on sleep duration across circadian time (ZT0–24) in wild-type flies.**

| ZT | Comparison | Control N | Dose N | Control mean | Dose mean | Diff(min) | p_value |
| --- | --- | --- | --- | --- | --- | --- | --- |
| 2 | 0.5 vs 0 | 15 | 15 | 12.73 | 1.47 | -11.26 | 0.019 |
| 2.5 | 0.5 vs 0 | 15 | 15 | 18.73 | 4.73 | -14 | 0.004 |
| 3.5 | 0.5 vs 0 | 15 | 15 | 20.07 | 9.4 | -10.67 | 0.039 |
| 4 | 0.5 vs 0 | 15 | 15 | 21.27 | 8.87 | -12.4 | 0.003 |
| 4.5 | 0.5 vs 0 | 15 | 15 | 23.13 | 8.8 | -14.33 | 0.002 |
| 8 | 0.5 vs 0 | 15 | 15 | 25 | 13.47 | -11.53 | 0.007 |
| 8.5 | 0.5 vs 0 | 15 | 15 | 20.93 | 10.93 | -10 | 0.020 |
| 8.5 | 1 vs 0 | 15 | 16 | 20.93 | 12.13 | -8.80 | 0.043 |
| 9.5 | 0.5 vs 0 | 15 | 15 | 19.33 | 8.07 | -11.26 | 0.035 |
| 10 | 0.5 vs 0 | 15 | 15 | 19.27 | 6.07 | -13.2 | 0.001 |
| 10 | 1 vs 0 | 15 | 16 | 19.27 | 9 | -10.27 | 0.011 |
| 12.5 | 0.5 vs 0 | 15 | 15 | 19.13 | 7.6 | -11.53 | 0.027 |
| 13 | 0.5 vs 0 | 15 | 15 | 26.47 | 13 | -13.47 | 0.000 |

**Table 6. Effects of simvastatin on locomotor activity across circadian time (ZT0–24) in wild-type flies.**

| ZT | Comparison | Control N | Dose N | Control mean | Dose mean | Diff(min) | p_value |
| --- | --- | --- | --- | --- | --- | --- | --- |
| 0 | 0 vs 1 | 15 | 16 | 30.2 | 53.06 | 22.86 | 0.0356 |
| 1 | 0 vs 1 | 15 | 16 | 26.2 | 50.12 | 23.92 | 0.00423 |
| 2.5 | 0 vs 0.5 | 15 | 15 | 11.2 | 31.67 | 20.47 | 0.00762 |
| 3.5 | 0 vs 0.5 | 15 | 15 | 8.6 | 24.67 | 16.07 | 0.0121 |
| 4 | 0 vs 0.5 | 15 | 15 | 6.27 | 24.73 | 18.46 | 0.00496 |
| 4.5 | 0 vs 0.5 | 15 | 15 | 7.8 | 24.07 | 16.27 | 0.0182 |
| 8 | 0 vs 0.5 | 15 | 15 | 4.6 | 20.13 | 15.53 | 0.011 |
| 8.5 | 0 vs 0.5 | 15 | 15 | 7.53 | 21.33 | 13.8 | 0.0285 |
| 8.5 | 0 vs 1 | 15 | 16 | 7.53 | 26.75 | 19.22 | 0.00121 |
| 9 | 0 vs 1 | 15 | 16 | 11.13 | 30.81 | 19.68 | 0.00443 |
| 10 | 0 vs 0.5 | 15 | 15 | 11.27 | 26.33 | 15.06 | 0.0497 |
| 10 | 0 vs 1 | 15 | 16 | 11.27 | 34.5 | 23.23 | 0.001 |
| 11 | 0 vs 1 | 15 | 16 | 33.8 | 54 | 20.2 | 0.0394 |
| 11.5 | 0 vs 1 | 15 | 16 | 37.07 | 65.25 | 28.18 | 0.00329 |
| 12 | 0 vs 1 | 15 | 16 | 55.93 | 87.38 | 31.45 | 0.00357 |
| 12.5 | 0 vs 0.5 | 15 | 15 | 17.07 | 42.13 | 25.06 | 0.0498 |
| 12.5 | 0 vs 1 | 15 | 16 | 17.07 | 48.88 | 31.81 | 0.00817 |
| 13 | 0 vs 0.5 | 15 | 15 | 4.27 | 27.93 | 23.66 | 0.00921 |
